# Physical Activity and Quality of Sleep in Patients with End-Stage Renal Disease on Hemodialysis: A Preliminary Report

**DOI:** 10.1155/2020/6918216

**Published:** 2020-08-26

**Authors:** Vasiliki Theodorou, Eleni Karetsi, Zoe Daniil, Konstantinos I. Gourgoulianis, Vasileios T. Stavrou

**Affiliations:** Laboratory of Cardio-Pulmonary Testing and Pulmonary Rehabilitation, Department of Respiratory Medicine, Faculty of Medicine, University of Thessaly, Larissa, Greece

## Abstract

Chronic kidney disease significantly impairs patients' daily lives and worsens their quality of life. The aim of this study was to investigate the physical activity and quality of sleep, during three days (previous day of dialysis, on the day of dialysis and after day of dialysis), in patients with end-stage renal on hemodialysis. 12 hemodialysis patients were included in our study, answered the Pittsburgh Sleep Quality Index (PSQI) questionnaire, and for each patient were used a smart bracelet for three days (day-pre- and posthemodialysis and day at hemodialysis) to record daily physical activity (steps, distance) and estimate the quality of sleep. Results showed differences between three days average of steps and distance and PSQI parameters “…engaging in social activity?” (steps, *p* = 0.006, distance, *p* = 0.006) and “…enthusiasm to get things done?” (steps, *p* = 0.029, distance, *p* = 0.030). Our study suggests interrelationship between sleep quality and physical activity.

## 1. Introduction

The kidneys exert a variety of actions on the body such as excretory, metabolic, and endocrine. They play a key role in regulating the concentrations of the constituents and excrete various metabolic by-products in the urine, preventing them from accumulating in the body [1]. Chronic kidney disease describes the condition in which the kidneys have been damaged so they cannot filter blood as effectively as healthy kidneys. It is characterized by the progressive decline in renal function and progresses from early stages (I and II) to intermediate stages (III and IV) and finally develops renal failure (Stage V) [2]. A significant proportion of patients have a first-degree relative of the disease [3], and the treatments of patients are extraperitoneal clearance (loss 85-90% of renal function) and kidney transplantation [4, 5]. The hemodialysis (3 to 5 hours and 3 times per week) process ensures the survival of patients and is the most widely used method of renal replacement, and it is based on the passage of large amounts of blood through thin tubes that are continuously immersed in solutions [6]. At the same time, the intense fatigue associated with the reduced energy the patient feels causes him to give up on several of his previous activities, and it is quite difficult to maintain an earlier life planning [7].

The quality of sleep is predetermined for respondents and may have been associated with symptoms of sleep disorders. Sleep breathing disorder is a condition for several chronic conditions in which partial or complete disruption of breath occurs many times throughout the night, resulting in daytime sleepiness or fatigue, limiting the functional ability of the individual and reduces his quality of life [8]. Moreover, the fear of death as well as the negative progression of the disease are of great concern to patients who develop sleep apnea [9] with predominant disorder, insomnia [1]. It is therefore unable to meet its basic needs, which puts additional stress on it, disrupting sleeping conditions [9]. Patients with chronic kidney disease has low physical condition with main contributing factors, respiratory and cardiovascular malfunctions, and limitations in peripheral muscles [10, 11].

The aim of this study was to investigate the physical activity and quality of sleep, during three days (previous day of dialysis, on the day of dialysis and after day of dialysis), in patients with end-stage renal on hemodialysis. We hypothesized that hemodialysis could affect physical activity and quality of sleep.

## 2. Materials and Methods

### 2.1. Participants

Twelve patients who were sent to the Hemodialysis Unit (Synchroni Polykliniki Larissas) between February 2019 and June 2019 constituted the initial study cohort (Table 1). Inclusion criteria were diagnosed chronic renal failure and hemodialysis for more than 2 years, age above 30 years, body mass index ≤ 35 kg/m^2^ and physical activity ≤ 150 minutes per week with heart rate ≥ 60% of predicted.

### 2.2. Data Collection

For each patient, anthropometric and morphological characteristics were recorded (Table 1), and they answered the Pittsburgh Sleep Quality Index (PSQI) questionnaire. Each patient used a smart bracelet (HUAWEI Honor Band 4, Shenzhen, China) for three days (day-pre- and posthemodialysis and day at hemodialysis) to record daily physical activity (steps, distance) and estimate the quality of sleep (sleep duration, REM and non-REM sleep stage, and total of awakening during sleep) accordingly. The recording days were Monday, Tuesday, and Wednesday.

### 2.3. Statistical Analysis

All variables were tested for normal distribution with the Kolmogorov-Smirnov and Shapiro-Wilk tests. Friedman and Kendall's nonparametric tests were used in cases of regularity violations. In the case of a normality approach, the test of equality of variances with Levene's test was used, and Student's (st-test) and ANOVA tests were used. Visual inspection of the relevant box plots for the existence of extreme values was done. Pearson's correlation was used to investigate the possible differences between control parameters. The level of statistical significance was set at *p* < 0.05. The statistical package used was IBM SPSS V23. (SPSS Inc., Chicago, Illinois, USA).

## 3. Results

### 3.1. Physical Activity

**Figure 1:**
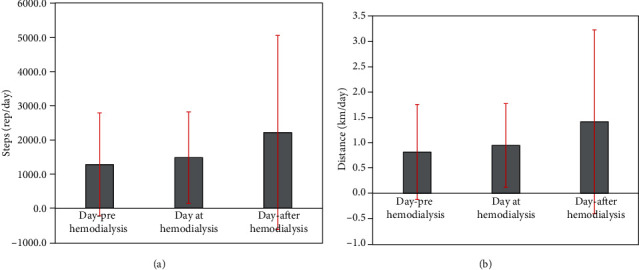
Physical activity results in patients with end-stage renal disease on hemodialysis among three days in steps (a) and distance (b).

Physical activity results did not show difference among three days in parameters steps (HEM_preday_: 1277.9 ± 1506.0, HEM_day_: 1477.0 ± 1341.9, and HEM_postday_: 2213.5 ± 2841.0, rep/day, *p* > 0.005) and distance (HEM_preday_: 0.82 ± 0.94, HEM_day_: 0.95 ± 0.83, and HEM_postday_: 1.41 ± 1.82, km/day, *p* > 0.005). Results showed asymmetry (2, 24), as well as the extremely high coefficient of variation (cv) (*x* = 1.17), leads to the conclusion of a completely nonhomogeneous distribution among the subjects of the study indicating a strong difference in patients' lifestyles. (Figure 1). From participants' physical activity on coverage distance after comparison between means and across distributions, finding the asymmetry factor (2, 38) and the high coefficient of variation (cv) (*x* = 1.15) leads to an enhanced view on the strong difference in physical activity of patients (Figure 1).

### 3.2. Sleep Quality

Results did not show different among three days in parameters of sleep quality (sleep duration: HEM_preday_: 283.1 ± 125.2, HEM_day_: 316.6 ± 182.2, and HEM_postday_: 285.3 ± 125.3, min, *p* > 0.005; REM sleep stage: HEM_preday_: 212.3 ± 99.6, HEM_day_: 236.3 ± 144.0, and HEM_postday_: 215.0 ± 101.8, min, *p* > 0.005; non-REM sleep stage: HEM_preday_: 63.8 ± 34.8, HEM_day_: 81.9 ± 59.8, and HEM_postday_: 64.6 ± 31.6, min, *p* > 0.005; and awakening frequency during sleep: HEM_preday_: 15.4 ± 21.9, HEM_day_: 10.8 ± 26.1, and HEM_postday_: 18.6 ± 31.0, rep, *p* > 0.005).

**Figure 2:**
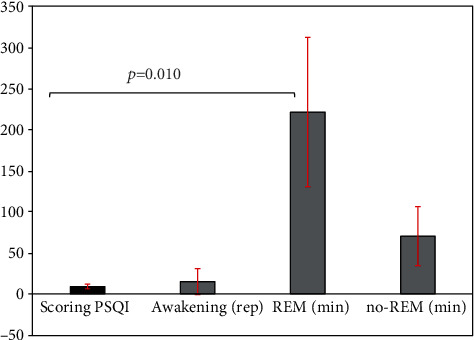
Sleep quality results in patients with end-stage renal disease on hemodialysis.

Sleep quality as assessed by PSQI showed differences in scoring (8.8 ± 2.8) and the three days' average values of the REM sleep stage (221.2 ± 90.8 min, *p* = 0.010, Figure 2). The other parameters of PSQI were not different in the estimated parameters of sleep quality with the use of smartwatch (Figure 2).

### 3.3. Sleep Quality and Physical Activity

Results showed differences between three days of average of steps and distance and PSQI parameters “*…*engaging in social activity?” (steps: *r* = 0.743, *p* = 0.006, distance: *r* = 0.744, *p* = 0.006) and “*…*enthusiasm to get things done?” (steps: *r* = 0.627, *p* = 0.029, distance: *r* = 0.626, *p* = 0.030). Moreover, results of the PSQI showed high values in patients with hemodialysis in parameters “wake up in the middle of the night or early morning,” “have pain,” and “cough or snore loudly” during the past month (Table 2).

## 4. Discussion

Chronic kidney failure is a disease that overwhelms the patient's body, especially at its final stage. The effect of the disease depends on the general physical condition and lifestyle of each patient. According to our results, there appeared to be no differences in physical activity at the time points before, during, and after dialysis. In particular, there was no clear indication that there were differences in the number of steps and distances between the time points before, during, and after dialysis in the patient population.

Dialysis is a therapeutic approach that involves the patient staying in the hospital unit or hemodialysis center for hours daily and therefore expected to affect their ability to do physical activity. The most systematic reviews and meta-analyses have included physical exercise programs mainly as supervised training performed at the treatment center, i.e., during inter- or intradialysis sessions [12]. According to Mallamaci et al. [12], exercise suggests a beneficial effect on physical performance and health endpoints which are achieved by improvement in aerobic and walking capacity. The results of the survey (HEM_preday_: 0.82 ± 0.94, HEM_day_: 0.95 ± 0.83, and HEM_postday_: 1.41 ± 1.82, km/day) are not in line with those of Brown et al. [7] who reported a decrease in patients' physical activity after hemodialysis. However, the findings of the research are in line with those of Bonner et al. [13] who showed the strong influence of the disease on work, daily life, and in general the lifestyle of patients while at the same time identifying differences between them due to their different lifestyles.

Hemodialysis patients often have sleep disorders due mainly to the stress and stress they experience due to the disease and the therapeutic approach. The inability to maintain sleep in the form of alarms is also found in the study by Artz et al. [9] which suggests that insomnia is the predominant disorder, followed by difficulty in starting to sleep, inability to maintain it, and restless sleep. In addition, the patient's need to comply with a rigorous treatment regimen such as dialysis creates a strong emotional burden and disrupts his sleep causing severe inability to initiate or even wake up. Their performance in physical activities further aggravates the anxiety of these patients and their physical and mental burden by affecting the quality of sleep as evidenced by the research of Kwabena and Awuah [1]. According to Roumelioti et al. [14], dialysis dependency was associated with poor sleep quality, and hemodialysis patients with sleep-disordered breathing were found to have poor sleep quality compared with those not on hemodialysis [15]. Our results showed that patients were classified as bad sleepers at the total PSQI score (8.8 ± 2.8) compared to previous studies which classified patients as poor sleepers when having score > 5.5 in PSQI [16]. According to Stavrou et al. [17, 18], the exercise, in patients with sleep disorders, may reduce the apnea-hypopnea index, improve the sleep quality during the daily physical activity, and may have a protective role in disease progression.

Concerns about the physical health and dependence of the artificial kidney machine cause severe symptoms of anxiety and depression in patients undergoing dialysis following the onset of mental disorders. Significant mental disorders identified are phobias and coercion in the therapeutic approach, while social disorders are not uncommon [13]. The change in appearance and the self-esteem decline that accompany it lead to introversion and isolation, and relationships with the social environment are affected. Problems occur mainly as daily frictions, and the patient's emotional state is particularly vulnerable. Social activities are significantly diminished, and failure to participate in daily activities and lack of interest are strongly observed. As the disease progresses, the individual is shown to be more irritable and nervous with severe behavior problems [19]. Abstinence from professional activities is also an important parameter in patients' lives, and their inability to meet their obligations creates feelings of impotence. The requirement to stay in the artificial kidney for several hours daily does not obviously allow patients to work normally and fall short of even light weight work capacity [7].

## 5. Limitations

In our study, there were some limitations. The low number of participants might be a statistical bias in our conclusions. Moreover, the nocturnal polysomnography, as a gold standard for the diagnosis of sleep-disordered breathing factor that influences patients sleep, was not evaluated.

## 6. Conclusions

Chronic kidney disease is a disease that has a profound impact on patients' daily lives. In particular for end-stage patients, the reduction of renal function and the treatment of renal failure induced by dialysis treatment strongly influence their daily lives and lead to a change in their lifestyle. Their daily habits change, as do the physical activities they can now perform, thereby causing changes in their daily lives and their sleep requiring them to adhere to a particularly rigorous treatment plan.

## Figures and Tables

**Table 1:** Patients characteristics. Continuous variables are presented as mean ± standard deviation.

		Mean ± SD
Age	yrg	63.8 ± 15.8
Gender (male)	%	75
Lean body mass	kg	61.7 ± 8.6
Body mass index	kg/m^2^	29.3 ± 4.6
Body surface area	m^2^	2.1 ± 0.5
Total body water	kg	44.0 ± 6.8
Hemodialysis	yrs	5.0 ± 5.3

**Table 2:** Relationship between PSQI results and three days average of steps and distance. Continuous variables are presented as mean ± standard deviation.

		Hemodialysis patients	Three days average of	
			Steps	Distance
1	When have you usually gone to bed?	2.2 ± 20.8	0.463	0.685
2	How long has it taken you to fall asleep each night?	18.3 ± 15.7	0.327	0.657
3	What time have you usually gotten up in the morning?	6.2 ± 1.1	0.235	0.458
4	How many hours of actual sleep did you get at night?	5.3 ± 1.8	0.126	0.215
5	Cannot get to sleep within 30 minutes	0.9 ± 1.0	0.319	0.426
6	Wake up in the middle of the night or early morning	1.3 ± 1.2	0.561	0.326
7	Have to get up to use the bathroom	0.8 ± 1.0	0.417	0.815
8	Cannot breathe comfortably	1.0 ± 1.0	0.138	0.152
9	Cough or snore loudly	1.3 ± 1.1	0.623	0.421
10	Feel too cold	1.0 ± 0.7	0.891	0.902
11	Feel too hot	1.1 ± 0.8	0.789	0.657
12	Have bad dreams	0.8 ± 0.6	0.092	0.101
13	Have pain	1.6 ± 0.5	0.119	0.093
During the past month…				
14	…how often have you taken medicine to help you sleep?	0.8 ± 1.0	0.637	0.685
15	…how often have you had trouble staying awake while driving, eating meals, or engaging in social activity?	0.5 ± 0.7	0.006	0.006
16	…how much of a problem has it been for you to keep up enthusiasm to get things done?	0.7 ± 0.9	0.029	0.030
17	…how would you rate your sleep quality overall?	1.2 ± 0.8	0.465	0.576

Questions 5-13 (scale: not during the past month (0), less than once a week (1), once or twice a week (2), and three or more times a week (3)); questions 14-17 (scale: very good (0), fairly good (1), fairly bad (2), and very bad (3)).

## Data Availability

The data used to support the findings of this study are available from the corresponding author upon request.
